# Mandibular range of motion in children with juvenile idiopathic arthritis with and without clinically established temporomandibular joint involvement and in healthy children; a cross-sectional study

**DOI:** 10.1186/s12969-021-00583-5

**Published:** 2021-07-03

**Authors:** Willemijn F. C. de Sonnaville, Caroline M. Speksnijder, Nicolaas P. A. Zuithoff, Daan R. C. Verkouteren, Nico W. Wulffraat, Michel H. Steenks, Antoine J. W. P. Rosenberg

**Affiliations:** 1 Department of Oral and Maxillofacial Surgery and Special Dental Care, University Medical Center Utrecht, Utrecht University, Heidelberglaan 100, 3584 CX Utrecht, PO Box 85500, The Netherlands; 2 Julius Center for Health Sciences and Primary Care, University Medical Center Utrecht, Utrecht, The Netherlands; 3 Department of Pediatric Rheumatology and Immunology, Wilhelmina Children’s Hospital, University Medical Center Utrecht, Utrecht University, Utrecht, The Netherlands

**Keywords:** Mandibular range of motion, Mouth opening, Arthritis, juvenile, Temporomandibular joint, Children, Cross-sectional study

## Abstract

**Background:**

Recognition of temporomandibular joint (TMJ) involvement in children with juvenile idiopathic arthritis (JIA) has gained increasing attention in the past decade. The clinical assessment of mandibular range of motion characteristics is part of the recommended variables to detect TMJ involvement in children with JIA.

The aim of this study was to explore explanatory variables for mandibular range of motion outcomes in children with JIA, with and without clinically established TMJ involvement, and in healthy children.

**Methods:**

This cross-sectional study included children with JIA and healthy children of age 6–18 years. Mandibular range of motion variables included active and passive maximum interincisal opening (AMIO and PMIO), protrusion, laterotrusion, dental midline shift in AMIO and in protrusion. Additionally, the TMJ screening protocol and palpation pain were assessed. Adjusted linear regression analyses of AMIO, PMIO, protrusion, and laterotrusion were performed to evaluate the explanatory factors. Two adjusted models were constructed: model 1 to compare children with JIA and healthy children, and model 2 to compare children with JIA with and without TMJ involvement.

**Results:**

A total of 298 children with JIA and 169 healthy children were included. Length was an explanatory variable for the mandibular range of motion excursions. Each centimeter increase in length increased AMIO (0.14 mm), PMIO (0.14 mm), and protrusion (0.02 mm). Male gender increased AMIO by 1.35 mm. Having JIA negatively influenced AMIO (3.57 mm), PMIO (3.71 mm), and protrusion (1.03 mm) compared with healthy children, while the discrepancy between left and right laterotrusion raised 0.68 mm. Children with JIA and TMJ involvement had a 8.27 mm lower AMIO, 7.68 mm lower PMIO and 0.96 mm higher discrepancy in left and right laterotrusion compared to healthy children.

**Conclusion:**

All mandibular range of motion items were restricted in children with JIA compared with healthy children. In children with JIA and TMJ involvement, AMIO, PMIO and the discrepancy between left and right laterotrusion were impaired more severely. The limitation in protrusion and laterotrusion was hardly clinically relevant. Overall, AMIO is the mandibular range of motion variable with the highest restriction (in millimeters) in children with JIA and clinically established TMJ involvement compared to healthy children.

**Supplementary Information:**

The online version contains supplementary material available at 10.1186/s12969-021-00583-5.

## Background

In children with juvenile idiopathic arthritis (JIA) the temporomandibular joint (TMJ) can be affected. Following the International League of Associations for Rheumatology (ILAR) classification and the clinical Juvenile Arthritis Disease Activity Score (cJADAS), the pediatric rheumatologist is encouraged to examine the TMJ and to count this joint [1, 2]. In recent years, TMJ arthritis in children with JIA has received increasing attention in research and clinical settings. Magnetic resonance imaging (MRI) is the gold standard to detect TMJ involvement. However, MRI has disadvantages such as the need for sedation and the need for infusion due to the contrast [3, 4]. As a result, there is more attention for orofacial examination and the recognition of TMJ involvement in children with JIA [5]. One of the recommendations for detection of TMJ dysfunction is assessment of the mandibular range of motion variables [5]. This includes assessments such as active and passive maximum interincisal mouth opening, dental midline deviation at maximum interincisal opening (MIO), protrusion, and laterotrusion [6]. In children with JIA, MIO is the most used variable to assess mandibular range of motion [5]. Mandibular movement characteristics are well-known variables that reflect the functional status of the masticatory system [5]. In children with JIA, researchers have reported a reduced MIO compared with healthy children [5, 7, 8]. A reduced mouth opening has also been reported as an indicator for TMJ arthritis in children with JIA [9, 10]. In many studies, a restricted mouth opening in children with JIA is defined by one overall cut-off value, without corrections for variables such as age, length, or gender [10–13]. However, a large variation among age groups in maximum mouth opening in healthy children has been reported, implying that the mouth opening can differ for each individual [14]. As a consequence, the utility of such an overall cut-off value for reduced mouth opening encompassing all ages would be low [14]. There is a need for individual-based information to establish reductions in mandibular range of motion excursions [15]. For this reason, the first aim of this study was to find explanatory demographic variables for mandibular range of motion outcomes in children. The second aim was to compare mandibular range of motion outcomes in children with JIA, with and without clinically established TMJ involvement, and in healthy children.

## Methods

This cross-sectional study was performed in children with JIA, aged 6 to 18 years, between January 2018 and February 2020 at the outpatient clinic of the Department of Pediatric Immunology and Rheumatology in collaboration with the Department of Oral and Maxillofacial Surgery and Special Dental Care of the University Medical Center (UMC) Utrecht, the Netherlands. The inclusion criteria for participation were children with JIA classified according to the ILAR criteria and aged between 6 and 18 years old. The children with JIA were in regular care, and not selected on TMJ function. Exclusion criteria were: 1) a history of mandibular trauma, 2) previous TMJ treatment, such as physical therapy, occlusal splints, intra-articular injection or maxillofacial surgery and 3) an additional orofacial condition not related to JIA (e.g., dental pain or a pre-existing jaw or temporomandibular disorder (TMD)). The measurements were carried out immediately after the regular consultation with the pediatric rheumatologist.

Healthy children were recruited from primary schools in Utrecht and a high school in Tilburg, the Netherlands, between February 2018 and April 2019. The inclusion and exclusion criteria for children with JIA were likewise applied for healthy children. In addition, healthy children with a TMJ screening protocol score ≥ 2 were excluded (*n* = 12) [11]. All measurements were conducted at the participating schools. The study protocol, with study ID NL.METC-17-528/C, was approved by the Ethics Committees of the UMC Utrecht on August 11th 2017. All participants and their parents and/or guardians received written information and provided their oral and signed informed consent.

Data extracted from the electronic medical records of the included children with JIA were: JIA subtype (see also Table 1), date of JIA diagnosis, medication, length, weight, gender, age, the presence of antinuclear antibody (ANA) or rheumatoid factor (RF), and the Clinical Juvenile Arthritis Disease Activity Score (cJADAS) [2]. Data collection was performed using the good clinical practice (GCP) compliant Electronic Data Capture (EDC) system Research Online. The proprietary EDC system is owned by the Julius Center at the UMC Utrecht.

**Table 1:** Demographics and clinical characteristics in children with JIA and in healthy children

	JIA *n* = 298	Healthy children *n* = 169	*P*-value	JIA with TMJ involvement *n* = 92	JIA without TMJ involvement *n* = 206	*P*-value
Gender (n, %)			0.000^a^			0.479^a^
Male	96 (32.2)	88 (52.1)		27 (29.3)	69 (33.5)	
Female	202 (67.8)	81 (47.9)		65 (70.7)	137 (66.5)	
Mean age (years; mean, SD)	12.7 (3.5)	11.5 (3.5)	0.000^b^	15.0 (4.0)	13.0 (6.0)	0.000^b^
Mean weight (kg; mean, SD)	50.7 (17.7)	46.9 (17.3)	0.026^b^	56.5 (24.5)	48.4 (28.2)	0.001^b^
Mean length (cm; mean, SD)	157.1 (18.3)	153.0 (20.9)	0.032^b^	165.0 (22)	160.5 (27.0)	0.001^b^
Orthodontic treatment (n, %)	48 (16.1)	17 (10.1)	0.070^a^	16 (17.4)	32 (15.5)	0.687^a^
Medication use (n, %)	225 (75.5)	14 (8.3)	0.000^a^	76 (82.6)	149 (72.3)	0.074^a^
Clinical remission off medication	73 (24.5)			16 (17.4)	55 (26.7)	0.000^a^
JIA subtype (n, %)						0.615^a^
Systemic	29 (9.7)			11 (12.0)	18 (8.7)	
Oligoarticular, persistent	113 (24.2)			26 (28.3)	87 (42.2)	
Oligoarticular, extended	32 (6.9)			11 (12.0)	21 (10.2)	
Polyarticular, RF-	63 (13.5)			24 (26.1)	39 (18.9)	
Polyarticular, RF+	15 (3.2)			5 (5.4)	10 (4.9)	
Enthesitis-related	18 (3.9)			6 (6.5)	12 (5.8)	
Psoriatic arthritis	15 (3.2)			5 (5.4)	10 (4.9)	
Undifferentiated	13 (4.4)			4 (4.3)	9 (4.4)	
Laboratory studies (n, %)						
Positive ANA	96 (20.6)			67 (32.5)	29 (31.5)	0.645^a^
Positive RF	17 (3.6)			12 (5.8)	5 (5.4)	0.457^b^
Positive HLA-B27	22 (4.7)			15 (7.3)	7 (7.6)	0.629^a^
Mean disease duration (months; mean, SD)	62.1 (51.2)			73.5 (56.9)	57.1 (47.8)	0.002^b^
cJADAS (n, %)						0.000^a^
0-2 (low)	189 (63.4)			49 (53.3)	140 (68.0)	
3-7 (moderate)	61 (20.5)			20 (21.7)	41 (19.9)	
≥8 (high)	42 (14.1)			19 (20.7)	23 (11.2)	
Missing	6 (2.0)			4 (4.3)	2 (1.0)	
Medication use (n, %)						
NSAIDS	87 (29.2)			33 (35.9)	54 (26.2)	0.090^a^
Corticosteroids	15 (5.0)			8 (8.7)	9 (4.2)	0.050^a^
DMARDS	138 (46.3)			43 (46.7)	95 (46.2)	0.627^a^
Biologicals	88 (29.5)			37 (40.2)	51 (24.8)	0.007^a^
No medication	73 (24.5)			16 (17.4)	55 (26.7)	0.074^a^
DMARDS (n, %)						0.404^a^
Methotrexate	119 (39.9)			33 (35.9)	86 (41.7)	
Leflunomide	12 (4.0)			6 (6.5)	6 (2.9)	
Azathioprine	2 (0.7)			1 (1.1)	1 (0.5)	
Sulphasalazine	2 (0.7)			1 (1.1)	1 (0.5)	
Other	3 (1.0)			2 (2.2)	1 (0.5)	
No DMARDS	160 (53.7)			49 (53.3)	111 (53.9)	
Biologicals (n, %)						0.073^a^
Adalimumab	42 (14.1)			14 (15.2)	28 (13.6)	
Etanercept	23 (7.7)			10 (10.9)	13 (6.3)	
Tocilizumab	6 (2.0)			3 (3.3)	3 (1.5)	
Canakinumab	5 (1.7)			2 (2.2)	3 (1.5)	
Golimumab	5 (1.7)			4 (4.3)	1 (0.5)	
Abatacept	1 (0.3)			1 (1.1)	0	
Anakinra	2 (0.7)			1 (1.1)	1 (0.5)	
Infliximab	1 (0.3)			0	1 (0.5)	
Other	3 (1.0)			2 (2.2)	1 (0.5)	
No Biologicals	210 (70.5)			55 (59.8)	155 (75.2)	
Items of the TMJ protocol score						
*History:*						
Problems in chewing (n, %)	42 (14.1)	0 (0.0)	0.000^a^	40 (43.5)	2 (1.0)	
Eating slower than others (n, %)	28 (9.4)	5 (3.0)	0.005^a^	26 (28.3)	2 (1.0)	
Biting hard food difficult (n, %)	38 (12.8)	0 (0.0)	0.000^a^	38 (41.3)	0 (0.0)	
Pain while eating (n, %)	54 (18.1)	0 (0.0)	0.000^a^	49 (53.3)	5 (2.4)	
Limited mouth opening (n, %)	33 (11.1)	1 (0.6)	0.000^a^	29 (31.5)	4 (1.9)	
*Examination*						
Limited mouth opening (n, %)	42 (14.1)	2 (1.2)	0.000^a^	33 (35.9)	9 (4.4)	
Crepitation (audible) (n, %)	33 (11.1)	0 (0.0)	0.000^a^	26 (28.3)	7 (3.4)	
Pain AMIO (n, %)	21 (7.0)	0 (0.0)	0.000^a^	20 (21.7)	1 (0.5)	
Deviation AMIO (>2 mm) (n, %)	51 (17.1)	1 (0.6)	0.000^a^	46 (50.0)	5 (2.4)	
*Inspection*						
Asymmetry (n, %)	57 (19.1)	13 (7.8)	0.001^a^	42 (45.7)	15 (7.3)	
Retrognathia (n, %)	23 (7.7)	9 (5.4)	0.325^a^	15 (16.3)	8 (3.9)	
Palpation pain						
TMJ (n,%)	28 (9.4)	4 (2.4)	0.004^a^	18 (19.6)	10 (4.9)	0.000^a^
Masseter muscle (n,%)	30 (10.1)	6 (3.6)	0.011^a^	22 (23.9)	8 (3.9)	0.000^a^
Temporal muscle (n,%)	13 (4.4)	0 (0.0)	0.006^a^	11 (12.0)	2 (1.0)	0.000^a^

*AMIO* active maximum interincisal mouth opening, *ANA* antinuclear antibody, *cJADAS* Clinical Juvenile Arthritis Disease Activity Score, *DMARDS* disease-modifying anti-rheumatic drugs, *HLA-B27* human leukocyte antigen B27, *JIA* juvenile idiopathic arthritis, *NSAIDS* non-steroidal anti-inflammatory drugs, *RF* rheumatoid factor, *SD* standard deviation, *TMJ* temporomandibular joint, ^‡^TMJ screening protocol score: Additional file A

^a^chi-squared test; ^b^independent sample t-test

### Assessments

In children with JIA and in healthy children, the mandibular range of motion variables and the TMJ, masseter muscle, and temporal muscle palpation pain were assessed. Moreover, the TMJ screening protocol score was established [11]. All participants in this study were examined by experienced examiners (WS, DV, MHS).

### Mandibular range of motion characteristics

Mandibular range of motion, deviation of the dental midlines ≥2 mm at maximum excursion, and pain provocation at maximum excursion in active maximum interincisal opening (AMIO) and passive maximum interincisal opening (PMIO) were assessed. Mandibular range of motion included the measurement of active and passive maximum interincisal mouth opening (AMIO and PMIO, respectively), protrusion, and left and right laterotrusion, followed by measuring the overjet and overbite. Mandibular range of motion measurements were recorded with a metal ruler to the nearest millimeter. The children were encouraged to open their mouths as wide as possible. The PMIO was assessed through the application of gentle stretch by the examiner, with the index finger and thumb on the incisal edges of the upper and lower incisors at the end of the active opening movement to increase the mouth opening. The AMIO and PMIO were measured between the incisal ridges of the upper and lower central incisors.

Protrusion was assessed by requesting the participants protrude the mandible as far anterior as possible. The horizontal distance between the upper central incisor and the lower central incisor was recorded with a ruler. Adding the overjet to this value produced the range of motion for protrusion. After correction for midline shift in occlusion, a deviation ≥2 mm of the midlines on maximum excursion was documented.

When measuring left and right laterotrusion, the dental midlines were used as reference points. In case of a midline shift in occlusion, a correction was carried out for the size of this shift (in millimeters). The difference between left and right laterotrusion with correction for a midline shift in occlusion was labeled “discrepancy between left and right laterotrusion.” The overjet and overbite were documented separately; overbite was not included in the mouth opening measurement. Limitation in condylar sliding was assessed by palpation of the TMJ region during AMIO and documented as limited or normal condylar sliding.

### TMJ and masseter and temporal muscle palpation pain

The TMJ and masseter and temporal muscles were palpated extra-orally for pain provocation. A numeric rating scale (NRS) was used in which no pain had a score of 0 and the worst imaginable pain scored 10. A NRS is a validated measurement tool for pain measurements [16]. When NRS_pain_ is > 3, the pain report is considered to be clinically relevant [17].

### TMJ screening protocol

To establish clinical TMJ involvement, we used the TMJ screening protocol [11]. The assessment of the TMJ status in children with JIA and in healthy children was carried out according to this protocol (Additional file 1). The sum of the history, examination, and inspection item scores (either 0 or 1) produced the TMJ screening protocol score.

The history items were collected by a questionnaire, adapted from the validated questionnaire “Screen” [18, 19]. All participants were interviewed following this questionnaire regarding their mandibular function. The history items, part of the TMJ screening protocol addressed: 1) problems in chewing, 2) eating slower than others, 3) difficulty in biting hard food, 4) pain while eating, and 5) a limited mouth opening.

The clinical examination items of the TMJ screening protocol addressed 1) AMIO, 2) crepitation during mouth opening and closing, 3) pain on AMIO, and 4) left or right mandibular midline deviation on opening wide [6].

The cut-off value for restricted mouth opening was ≤35 mm for children 10 years old and younger, and ≤ 40 mm in children older than 10 years [15]. A clinically visible deviation at maximum mouth opening (≥ 2 mm on maximum excursion) was scored, using a metal ruler as a reference line. Auscultation of the TMJ to establish crepitation was performed using a stethoscope during the opening and closing of the mouth. The stethoscope was placed on the skin over the TMJ. Patients were asked to open and close their mouth as far as possible.

The inspection items of the TMJ screening protocol originated from the same examination form and addressed 1) facial asymmetry and 2) retrognathia. Facial asymmetry comprised the mandibular ramus length and chin deviation. Ramus length was assessed by palpating the left and right mandibular angle simultaneously and comparing the left and right side. Differences in right and left ramus length yielded a positive score.

Retrognathia was evaluated by the examiner using the images of the TMJ screening protocol (Additional file 1). A retrognathic profile as in the image was assigned a positive score. A normal profile and a class II profile scored zero points.

Each positive item of the TMJ screening protocol received 1 point; negative scoring items received 0 points. All positive items produced the TMJ screening protocol score. A TMJ protocol score ≥ 2 has been suggested to indicate clinically established TMJ involvement in children with JIA [11].

### Statistical analysis

Categorical variables of the children are presented as numbers and percentages, while continuous variables are presented as means and standard deviations in the case of normally distributed variables. For the analyses of all clinical data (AMIO, PMIO, protrusion, laterotrusion left side, laterotrusion right side, discrepancy between left and right laterotrusion, overjet and overbite, TMJ and masticatory muscle pain, TMJ screening protocol score, and demographics), the unpaired Student’s t-test was used for continuous data, and the chi-squared test was used for dichotomous or ordered categorical outcomes. For the variables AMIO, PMIO, protrusion, and discrepancy between left and right laterotrusion, we performed adjusted analysis with corrections for length and gender (i.e., for each variable separately). Two adjusted models were made: model 1 to compare children with JIA and healthy children, and model 2 to compare children with JIA with TMJ involvement and without TMJ involvement.

In a secondary analysis, we explored the effect of disease characteristics, JIA subtype, cJADAS, medication use and orthodontic treatment with corrections for length and gender. The variable deviation MIO was not included in the adjusted models 1 and 2 because these variables are part of the TMJ screening protocol; therefore, they may indicate TMJ involvement. Model validity (i.e., normality, homoscedasticity) was assessed with residual analysis [20].

Age and length showed a high correlation (i.e., collinearity), suggesting the explanatory impact is very similar. The adjusted analysis for AMIO including length resulted in a slightly higher R-squared (*R*^*2*^ = 0.19; Table 4) compared with age (*R*^*2*^ = 0.18; Additional file 2 and 3). Therefore, we included length instead of age in the adjusted analysis. Because in the literature age is more commonly used, we also presented the adjusted analyses including age for AMIO, PMIO, protrusion and discrepancy between left and right laterotrusion in Appendices B and C. In addition, when we graphically evaluated the effect of age on AMIO and PMIO, we noticed a non-linear effect, suggesting that AMIO and PMIO reach their maxima during adolescence. We therefore include age squared in addition to a linear term in the unadjusted model. By contrast, the variable length seems to have a linear effect.

Results are reported as regression coefficients with 95% confidence intervals (CIs) and *p*-values. For categorical variables, regression coefficients represent the difference in mean mandibular range of motion (AMIO, PMIO, protrusion, and discrepancy between left and right laterotrusion). For continuous variables, the regression coefficient represents the increase in mandibular range of motion for each unit increase in the explanatory variable. A probability of less than 0.05 was accepted as significant. Tests were performed using SPSS 25 (IBM SPSS Statistics for Windows, Version 25.0. Armonk, NY: IBM Corp).

## Results

This study included 298 children with JIA and 169 healthy children; the demographic data are presented in Table 1. In the children with JIA, 202 were girls (67.8%), with a mean age of 12.7 years (standard deviation (SD) 3.5), a mean length of 157.1 cm (SD 18.3), and a mean disease duration of 62.1 months. In the healthy children, 81 were girls (47.9%), with a mean age of 11.5 years (SD 3.5) and a mean length of 153.0 cm (SD 20.9). Ninety-two (30.9%) out of 298 children with JIA had TMJ involvement (TMJ protocol score ≥ 2). Children with JIA and TMJ involvement were older (*p* = 0.032), had less clinical remission off medication (*p* = 0.000), a longer disease duration (*p* = 0.002), a higher cJADAS (p = 0.000), and used more corticosteroids (*p* = 0.050) and biologicals (*p* = 0.007; Table 1). TMJ pain and masseter and temporal muscle pain on palpation were all statistically significantly more prevalent in children with JIA compared with healthy children (*p* = 0.000). In children with JIA and TMJ involvement, these pain outcomes were more prevalent compared with children with JIA without TMJ involvement (*p* = 0.000).

### Mandibular range of motion outcomes

The mandibular range of motion data, not corrected for age and gender, are presented in Table 2. AMIO, PMIO, and protrusion were lower in children with JIA compared with healthy children (*p* = 0.000; Table 2). The discrepancy between left and right laterotrusion, midline deviation on AMIO and in protrusion, and condylar sliding were more often present in children with JIA (*p* = 0.000; Table 2), but not for laterotrusion left and laterotrusion right (Table 2). In children with JIA and TMJ involvement, AMIO and PMIO were lower 12.4% (5.9 mm) and 9.6% (4.7 mm), respectively, compared with children with JIA without TMJ involvement (*p* = 0.000; Table 2). In children with JIA and TMJ involvement compared with children with JIA without TMJ involvement, there was a higher prevalence for discrepancy between left and right laterotrusion (1.2 mm), deviation during AMIO (50.0%), during protrusion (33.7%; p = 0.000) and limited condylar sliding (15.2%; p = 0.000; Table 2). Overjet and overbite did not statistically differ between children with JIA and healthy children, and between children with JIA with and without clinically established TMJ involvement.

**Table 2:** Mandibular range of motion in children with JIA and in healthy children

	JIA (***n*** = 298)	Healthy (***n*** = 169)	***P-value***	JIA with TMJ involvement (***n*** = 92)	JIA without TMJ involvement (***n*** = 206)	***P-value***
AMIO (mm; mean, SD)	45.7 (7.6)	49.0 (6.1)	0.000^a^	41.6 (8.7)	47.5 (6.4)	0.000^a^
PMIO (mm; mean, SD)	47.4 (7.9)	50.4 (6.1)	0.000^a^	44.1 (8.7)	48.8 (7.0)	0.000^a^
Protrusion (mm; mean, SD)	7.5 (2.2)	8.5 (2.2)	0.000^a^	7.3 (2.4)	7.6 (2.2)	0.462^a^
Laterotrusion left (mm; mean, SD)	9.4 (2.1)	9.7 (1.6)	0.092^a^	9.1 (2.2)	9.6 (1.8)	0.032^a^
Laterotrusion right (mm; mean, SD)	9.4 (2.1)	9.7 (1.6)	0.044^a^	9.0 (2.3)	9.6 (1.8)	0.019^a^
Discrepancy between left and right laterotrusion (mm; mean, SD)	1.0 (1.3)	0.3 (0.5)	0.000^a^	1.2 (1.6)	0.6 (0.9)	0.000^a^
Overbite (mm; mean, SD)	2.5 (1.4)	2.3 (1.5)	0.127^a^	2.3 (1.3)	2.5 (1.4)	0.181^a^
Overjet (mm; mean, SD)	2.9 (1.7)	3.0 (1.4)	0.880^a^	3.0 (1.8)	2.9 (1.7)	0.509^a^
Deviation AMIO* (n, %)	51 (17.1)	1 (0.6)	0.000^b^	46 (50.0)	5 (2.4)	0.000^b^
Deviation protrusion* (n, %)	38 (12.8)	0 (0.0)	0.000^b^	31 (33.7)	7 (3.4)	0.000^b^
Limited condylar sliding (n, %)	17 (5.7)	1 (0.6)	0.006^b^	14 (15.2)	3 (1.5)	0.000^b^

^a^ independent sample t-test; ^b^ chi-squared test

*AMIO* active maximum interincisal opening; *cJADAS* Clinical Juvenile Arthritis Disease Activity Score; *DMARDS* disease-modifying antirheumatic drugs; *JIA* juvenile idiopathic arthritis; *NSAIDS* non-steroidal anti-inflammatory drugs; *PMIO* passive maximum interincisal opening; *SD*: standard deviation; *TMJ*: temporomandibular joint;

*Deviation AMIO and protrusion were defined as mandibular midline deviation during AMIO and mandibular midline deviation during protrusion

### Active maximum interincisal mouth opening

The adjusted linear regression models for AMIO demonstrated the following significant explanatory variables (*p* < 0.05, Table 3): age, age squared, length, male gender, cJADAS, medication use, deviation during AMIO and protrusion, TMJ palpation pain, temporal muscle palpation pain and limited condylar sliding. JIA subtype was not an explanatory variable for AMIO (*p* = 0.643).

**Table 3:** Adjusted linear regression for mandibular range of motion variables, with correction for length and gender

	AMIO		PMIO		Protrusion		Discrepancy in laterotrusion*	
Variable	Regression coefficients (95% CI)	***P-value***	Regression coefficients (95% CI)	***P-value***	Regression coefficients (95% CI)	***P-value***	Regression coefficients (95% CI)	***P-value***
Age	1.30 (0.62–1.98)	0.000	1.48 (0.77–2.19)	0.000	0.28 (−0.06–0.51)	0.014	−0.01 (− 0.13–0.10)	0.859
Age squared (centered at 6)	−0.06 (− 0.12 – − 0.00)	0.036	− 0.07 (− 0.13 – − 0.01)	0.018	−0.02 (− 0.04–0.00)	0.029	0.00 (− 0.01–0.01)	0.550
Length	0.13 (0.09–0.0.16)	0.000	0.13 (0.10–0.16)	0.000	0.01 (0.00–0.02)	0.039	0.00 (−0.00–0.01)	0.410
Male gender	2.09 (0.82–3.36)	0.001	1.52 (0.17–2.86)	0.027	0.36 (−0.06–0.79)	0.096	−0.28 (− 0.07–0.49)	0.009
JIA subtype	0.00 (−0.01–0.02)	0.643	0.00 (− 0.01–0.02)	0.674	0.00 (− 0.01–0.00)	0.849	− 0.00 (− 0.00–0.00)	0.488
cJADAS	−0.39 (− 0.57 – − 0.21)	0.000	− 0.37 (− 0.56 – − 0.18)	0.000	−0.04 (− 0.10–0.03)	0.237	0.02 (− 0.01–0.05)	0.178
Medication use	− 2.00 (− 3.24–0.71)	0.002	−1.89 (− 3.22 – − 0.55)	0.006	−0.65 (− 1.07 – − 0.23)	0.003	0.67 (0.44–0.84)	0.000
Deviation AMIO**	−6.00 (−7.90 – − 4.10)	0.000	−5.44 (−7.41– − 3.47)	0.000	− 1.15 (− 1.81 – − 0.48)	0.001	0.91 (0.59–1.22)	0.000
Deviation protrusion**	−5.80 (−8.00 – − 3.59)	0.000	−5.40 (− 7.69 – − 3.12)	0.000	−1.30 (− 2.07 – − 0.53)	0.001	0.96 (0.60–1.32)	0.000
TMJ palpation pain	− 4.93 (− 7.34 – − 2.52)	0.000	− 4.86 (− 7.34 – − 2.37)	0.000	−1.30 (− 2.11 – − 0.49)	0.002	0.17 (− 0.24–0.57)	0.419
Masseter muscle palpation pain	− 2.23 (− 4.57–0.12)	0.063	1.92 (−4.37–0.53)	0.125	−0.41 (− 1.20–0.37)	0.302	0.03 (− 0.36–0.43)	0.864
Temporal muscle palpation pain	− 5.82 (− 9.58–2.06)	0.002	− 5.12 (− 8.99 – − 1.24)	0.010	−1.81 (− 3.10 – − 0.52)	0.006	0.34 (− 0.29–0.96)	0.288
Limited condylar sliding	−9.38 (− 12.50 – − 6.27)	0.000	−9.70 (− 12.90 – -6.50)	0.000	− 2.27 (− 3.38 – -1.16)	0.000	1.86 (1.36–2.37)	0.000
Orthodontic treatment	− 0.57 (− 2.41–1.27)	0.545	0.34 (−2.28–1.59)	0.727	−0.27 (− 0.88–0.34)	0.389	0.24 (− 0.07–0.55)	0.128

*AMIO* active maximum interincisal opening; *CI* confidence interval; *JIA* juvenile idiopathic arthritis; *PMIO* passive maximum interincisal opening; *TMJ* temporomandibular joint

The adjusted model with corrections for length and gender showed the association of each variable to AMIO, PMIO, protrusion, and discrepancy in laterotrusion

*Discrepancy in laterotrusion was defined as the difference between left and right laterotrusion with correction for midline deviation. TMJ involvement is proposed as a TMJ protocol score ≥ 2 in JIA patients [5]

**Deviation AMIO and protrusion were defined as mandibular midline deviation during AMIO and mandibular midline deviation during protrusion

**Fig. 1:**
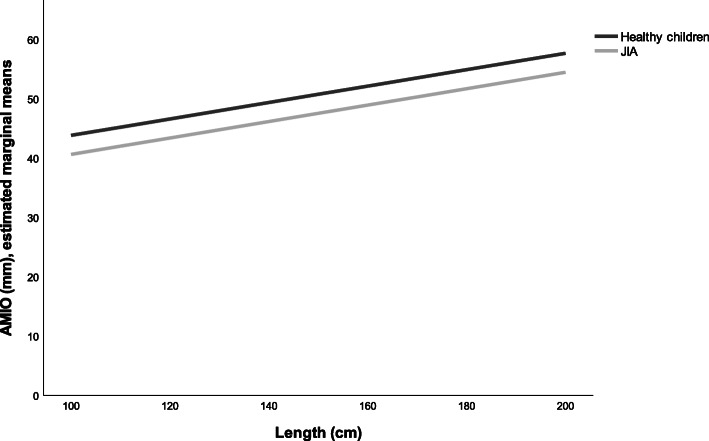
Estimated marginal means of AMIO in children with JIA and in healthy children

**Fig. 2:**
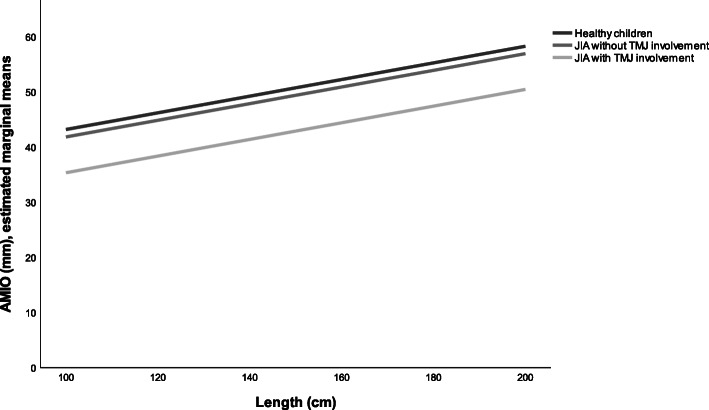
Estimated marginal means of AMIO in children with JIA with and without TMJ involvement

The adjusted analysis model 1 indicated 3.6 mm less AMIO (95% CI -4.9 – − 2.3, *p* = 0.000) in children with JIA compared with healthy children (Table 4, model 1, Fig. 1). When we included TMJ involvement in the adjusted analysis, the AMIO was 8.3 mm lower in children with JIA and TMJ involvement (Table 4, model 2, Fig. 2). In children with JIA without TMJ involvement, the AMIO was 1.6 mm less (95% CI -2.9 – − 0.3, *p* = 0.013) compared to healthy children. The adjusted variable male gender indicated 1.4 mm more AMIO (95% CI 2.6–0.1, *p* = 0.04); each centimeter in length increased AMIO by 0.1 mm (95% CI 0.1 – − 0.2, *p* = 0.000).

**Table 4:** Adjusted linear regression for mandibular range of motion in children with JIA and healthy children

**Model 1: Children with JIA vs healthy children**								
	**AMIO**		**PMIO**		**Protrusion**		**Discrepancy in laterotrusion:**	
Variable	**Regression coefficients** **(95% CI)**	***P-value***	**Regression coefficients** **(95% CI)**	***P-value***	**Regression coefficients** **(95% CI)**	***P-value***	**Regression coefficients** **(95% CI)**	***P-value***
JIA vs healthy children	−3.57 (− 4.85 – − 2.28)	0.000	−3.71 (− 5.09 – − 2.33)	0.000	−1.03 (1.46 – − 0.60)	0.000	0.68 (0.47–0.89)	0.000
Male gender	1.35 (2.61–0.09)	0.035	0.73 (− 0.61–2.06)	0.284	0.14 (− 0.29–0.56)	0.525	−0.15 (− 0.06–0.35)	0.171
Length	0.14 (0.10–0.17)	0,000	0.14 (0.11–0.18)	0.000	0.02 (0.01–0.03)	0.005	0.00 (−0.01–0.01)	0.905
Intercept	32.51 (27.31–37.71)		32.72 (27.29–38.14)		7.23 (5.47–9.00)		−0.54 (−1.41 – − 0.33)	
R^2^	0.19		0.18		0.06		0.10	
**Model 2: Children with JIA with vs without TMJ involvement**								
	**AMIO**		**PMIO**		**Protrusion**		**Discrepancy in laterotrusion:**	
Variable	**Regression coefficients** **(95% CI)**	***P-value***	**Regression coefficients** **(95% CI)**	***P-value***			**Regression coefficients** **(95% CI)**	***P-value***
JIA vs healthy children	−1.61 (−2.89 – −0.34)	0.013	−2.09 (−3.49 – − 0.70)	0.003	− 0.94 (− 1.40– -0.48)	0.000	0.58 (0.35–0.80)	0.000
JIA with vs without TMJ involvement	−6.67 (−8.17 – −5.14)	0.000	−5.59 (− 7.21 – −3.97)	0.000	− 0.31 (− 0.86–0.25)	0.283	0.36 (0.09–0.62)	0.008
Male gender	1.12 (−0.05–2.30)	0.060	0.53 (−0.75–1.80)	0.418	0.13 (−0.30—0.55)	0.564	−0.13 (− 0.34–0.08)	0.212
Length	0.15 (0.12–0.18)	0.000	0.16 (0.12–0.19)	0.000	0.02 (0.01–0.03)	0.004	0.00 (−0.01–0.01)	0.873
Intercept	28.29 (23.36–33.22)		29.07 (23.79–34.33)		7.04 (5.24–8.84)		−0.32 (−1.20–0.57)	
R^2^	0.30		0.26		0.07		0.11	

*AMIO* active maximum interincisal opening; *CI* confidence interval; *JIA* juvenile idiopathic arthritis; *PMIO* passive maximum interincisal opening; *TMJ* temporomandibular joint

*Discrepancy in laterotrusion was defined as the difference in laterotrusion (in millimeters) between the left and right side. TMJ involvement is proposed as a TMJ protocol score ≥ 2 in JIA patients

### Passive maximum interincisal mouth opening

The adjusted analysis for PMIO showed the following significant explanatory variables (Table 3): age, age squared, length, male gender, cJADAS, medication use, deviation during AMIO, deviation during protrusion, TMJ palpation pain, temporal palpation pain and limited condylar sliding.

In the adjusted model 1, PMIO was 3.7 mm less (95% CI: − 5.1 – − 2.3, *p* = 0.000) in children with JIA compared with healthy children (Table 4, model 1). When TMJ involvement was included in the adjusted model, PMIO was 5.6 mm lower (95% CI -7.2 – − 4.0, *p* = 0.000; Table 4, model 2). Overall, children with JIA and TMJ involvement had a 7.7 mm lower PMIO, while children with JIA without TMJ involvement had a 2.1 mm lower PMIO (Table 4, model 2) compared to healthy children. The adjusted variable length increased PMIO by 0.2 mm (95% CI 0.1–0.2, p = 0.000).

### Protrusion

The adjusted linear model indicated that the explanatory factors for protrusion were length, medication use, deviation during AMIO and protrusion, TMJ palpation pain, temporal muscle palpation pain and limited condylar sliding (*p* < 0.05; Table 3).

The adjusted model 1 compared children with JIA and healthy children and included the variables male gender (95% CI − 0.3–0.6, *p* = 0.525), length (95% CI 0.0–0.0, *p* = 0.005) and JIA (95% CI -1.5 – − 0.6, *p* = 0.000). The model showed 1.0 mm less protrusion in children with JIA compared with healthy children, and an increase of 0.01 mm for each centimeter in length (Table 4, model 1). Model 2 indicated that TMJ involvement did not influence protrusion significantly (95% CI − 0.9–0.3, *p* = 0.283).

### Discrepancy between right and left laterotrusion

The adjusted model indicated the following explanatory variables regarding the discrepancy between right and left laterotrusion: male gender, medication use, deviation during AMIO and protrusion and limited condylar sliding (p < 0.05; Table 3).

The adjusted model 1 showed the explanatory variable JIA and indicated JIA increased the discrepancy between right and left laterotrusion by 0.7 mm (95% CI 0.5–0.9, p = 0.000, Table 4, model 1). The adjusted model 2 included the explanatory variables JIA (95% CI 0.4–0.8, p = 0.000), TMJ involvement (95% CI 0.1–0.6, *p* = 0.008; Table 4), male gender (95% CI -0.3 – 0.1, *p* = 0.212), and length (95% CI − 0.0–0.0, *p* = 0.873). These results indicated that children with JIA and TMJ involvement had a 0.9 mm, and children with JIA without TMJ involvement had 0.6 mm more discrepancy between right and left laterotrusion compared to healthy children.

## Discussion

The results of this study demonstrated a restriction of all mandibular range of motion variables in children with JIA compared with healthy children. The variables AMIO and PMIO had the largest restriction (in millimeters) as a consequence of JIA. Protrusion and the discrepancy between left and right laterotrusion were negatively influenced by a minimally amount. In children with JIA and clinically established TMJ involvement, the reduction in AMIO, PMIO, and discrepancy between laterotrusion was more prominent. Other explanatory variables for AMIO were an increase by male gender and by length.

The mandibular range of motion variable with the greatest restriction in children with JIA was AMIO, in particular in children with JIA and TMJ involvement. This finding is in concordance with the literature, which has shown a reduced MIO in children with JIA and TMJ involvement [21–23]. In children with JIA without TMJ involvement, AMIO was 1.6 mm lower compared to healthy children. This minimal AMIO reduction is not clinically relevant, as it is well below the smallest detectable difference of 4.9 mm [24]. Therefore, our finding in children with JIA without TMJ involvement may not be clinically relevant. The discrimination between children with JIA with and without clinically established TMJ involvement also sheds light on the discussion as to whether children with JIA have a limited mouth opening compared with healthy children. Such a difference has not always been found [25]. Considering all children with JIA without separating the subgroups with and without clinically established TMJ involvement may have smoothed the differences we found (Table 4). Our TMJ screening protocol seems to demonstrate these “hidden” differences.

In the clinical evaluation of patients with TMDs, PMIO is used to distinguish articular and muscular origins. It has been stated that PMIO exceeds AMIO by 1–2 mm in individuals without TMD symptoms [26]. If PMIO is increased ≥3 mm compared with AMIO, a reduced mouth opening with a muscular component must be considered [27]. In our study, we measured both AMIO and PMIO and constructed adjusted linear regression models for both measurements (as shown in Table 4). The effect of JIA and TMJ involvement explained a 8.3 mm lower AMIO and a 7.7 mm lower PMIO. This difference between AMIO and PMIO is well below 2 mm, implying that the mouth opening reduction due to the variables JIA and TMJ involvement has as expected, a mainly articular background.

Various methods to measure MIO have been described, such as AMIO, PMIO, MIO including the overjet vs interincisal distance, and finger breadth [28]. In our study, the variables JIA and TMJ involvement led to a similar reduction in AMIO and PMIO. This finding implies that either way of measuring MIO may be applicable. The most used method for measuring MIO in children with JIA is AMIO without corrections for overbite [23]. In our opinion, in the context of screening this method is the most reliable approach and is applicable for the rheumatologist in clinical practice, because it is a quick and easy measuring tool for everyday practice [29].

Protrusion has rarely been used as a measurement in children with JIA [8, 23]. However, the international consensus recommendations advise using all mandibular range of motion variables to assess joint function in children with JIA [5]. In our study, we found a minimal restriction in protrusion between children with JIA and healthy children. However, we did not confirm a different protrusion in children with or without TMJ involvement, while the other mandibular range of motion variables were more impaired in children with JIA and TMJ involvement. This finding may imply that protrusion is not a mandibular mandibular range of motion variable that may reflect TMJ dysfunction in children with JIA and TMJ involvement. This may have to do with the condylar sliding trajectory during protrusion. The maximum trajectory reached when opening the mouth is larger than in protrusion. A reduction will therefore become manifest in protrusion less often than when opening the mouth. This might be the same for laterotrusion.

Other studies have indicated difficulties measuring protrusion in younger children and have pointed out that protrusion is less applicable in a clinical setting [30]. In our study, we had difficulties measuring the protrusion in 15 children, with a mean age of 9.2 years. Of a total of 476 individual measurements, in 3.1% of the measurements we were unable to measure the protrusion. We do not consider this prevalence as a prominent issue. Overall, we were able to measure protrusion in most children and conclude that measuring protrusion does not have an extra value to assess TMJ dysfunction in children with JIA and TMJ involvement.

Laterotrusion has hardly been studied in children with JIA. In one study, the researchers used a scoring system for clinical signs of TMJ involvement [31]. The results indicated that one of the signs of TMJ involvement that should be an alert for the clinician is laterotrusion ≤5 mm to either the left or right side. There were no data available of the exact measured laterotrusion in this study. Other studies have measured laterotrusion in children with JIA and in controls, but the authors reported no statistically significant differences between these groups [8, 25]. Based on our study, we can confirm these outcomes. However, in healthy individuals differences between left and right laterotrusion are expected to be zero; thus, we compared the left and right laterotrusion in each individual. The discrepancy between left and right laterotrusion was statistically more prevalent in children with JIA compared with healthy children. In addition, the adjusted model 2 showed a significant effect of JIA and TMJ involvement, although the effect was minimal: a 0.9 mm difference in children with JIA and TMJ involvement (Table 4). In a clinical setting, this small difference is hardly noticeable and therefore not clinically relevant. Children with bilaterally limited laterotrusion will not be detected through the variable ‘discrepancy between left and right laterotrusion’. Moreover, in case of a dental midline shift, the measurement is more complex and more time consuming. In case of a larger discrepancy, this finding will become apparent when assessing AMIO characteristics. Therefore, not including protrusion and laterotrusion in our screening protocol is supported by these findings. In the context of screening for TMJ involvement in children with JIA, these two measurements offer similar information as the assessment of AMIO characteristics.

The strengths of this study are the measurement of all mandibular range of motion variables, the comparison between children with JIA with and without TMJ involvement and healthy controls, and the large study population. A limitation is the use of the TMJ protocol to clinically establish TMJ involvement, instead of MRI as the “gold standard.” We did not use imaging techniques to establish TMJ arthritis; instead, we classified children with JIA with clinically established TMJ involvement on the basis of the TMJ screening protocol score [11]. We found a reduction in the mandibular range of motion variables in children with JIA without TMJ involvement, although reductions are small and hardly noticeable in a clinical setting. The decrease that we found compared to healthy children may be the effect of our clinical screening method for TMJ involvement. Our clinical establishment of TMJ involvement is probably less accurate than diagnosis per MRI [5, 31, 32]. Since the TMJ is quoted as the ‘silent’ joint, implicating that arthritis was not detected by clinical examination only [33, 34], the reduction of mandibular range of motion variables in children with JIA can therefore be related to underdiagnoses of TMJ arthritis. However, imaging in young children is a burden due to disadvantages such as the need for sedation, the need for infusion due to the contrast, the concern with contrast retention in the human brain, limited availability and expertise, as well as high costs [3, 4]. In case of patients with dental braces, scattering can also lead to inadequate images.

The TMJ protocol score comprises three items related to MIO. This could invalidate the adjusted linear regression model for AMIO and PMIO. To analyze the influence of these related variables for MIO, we constructed a TMJ screening protocol score without the items “limited mouth opening in the medical history,” “limited mouth opening during clinical examination,” and “deviation during active maximum interincisal opening (AMIO).” The corrected TMJ protocol scores were plotted and seemed to have a linear association with AMIO and PMIO. In addition, a corrected variable for TMJ involvement was derived from the corrected TMJ protocol score; a TMJ protocol score ≥ 2 was classified as TMJ involvement with correction for the items related to MIO. The corrected TMJ involvement variables were included in an unadjusted linear regression model for AMIO and PMIO (Additional files 4 and 5). We compared the model with the original TMJ involvement variable and the model with the corrected TMJ involvement variable. The unadjusted linear regression model for AMIO showed a regression coefficient for TMJ involvement of − 6.6 mm, compared with − 5.9 mm for the corrected TMJ involvement. The same analysis for PMIO showed a regression coefficient for TMJ involvement of − 5.4 mm, compared with − 4.9 mm for the correct TMJ involvement. The difference of 0.7 mm and 0.5 mm, respectively, seems to have minor clinical relevance in the context of clinical mandibular range of motion measurement, especially because the smallest detectable difference of 4.9 mm is mentioned for AMIO in literature [24]. Overall, we assume that the original variable for TMJ involvement in our analysis will lead to similar conclusions related to AMIO and PMIO.

This study indicated that AMIO is the most clinically relevant mandibular range of motion measurement in children with JIA and TMJ involvement. This finding is in line with former studies in the clinical temporomandibular working field [5]. In future research, it would be interesting to define a cut-off value for reduced AMIO for children with JIA with TMJ involvement by considering the relevant demographic variables. Other studies have documented a wide individual difference in MIO in healthy children [14, 28, 35]. We corrected for the individual variables gender and length. Another possible method to overcome the prominent individual variance is to investigate MIO in a longitudinal study and compare the patients with themselves [14]. Such a study may also shed light on the clinical relevance of following up on AMIO measurement as indicators for TMJ involvement and ultimately TMJ arthritis in children with JIA.

## Conclusions

The mandibular range of motion variables, as reflected in this study by AMIO, PMIO, protrusion, and discrepancy between left and right laterotrusion, were all impaired in children with JIA compared with healthy children. In children with JIA and TMJ involvement, the restriction was more severe for AMIO and PMIO and more accentuated for the discrepancy between left and right laterotrusion. A clinically relevant effect of having JIA and TMJ involvement on mandibular range of motion was mainly reflected in AMIO and PMIO. However, laterotrusion and protrusion were also significantly less in children with JIA, although the effect was around 1 mm and irrelevant in a clinical setting. The demographic variables length explained an increase in AMIO, PMIO, and protrusion. Male gender was related to a higher AMIO but not in the other mandibular range of motion variables.

## Supplementary Information

**Additional file 1.**

**Additional file 2.**

**Additional file 3.**

**Additional file 4.**

**Additional file 5.**

## Data Availability

The datasets used and/or analyzed during the current study are available from the corresponding author on reasonable request.

## Abbreviations

AMIO: Active maximum interincisal opening
ANA: Antinuclear antibody
CI: Confidence interval
cJADAS: Clinical juvenile arthritis disease activity score
DMARDS: Disease-modifying anti-rheumatic drugs
EDC: Electronic data capture
GCP: Good clinical practice
HLA-B27: Human leukocyte antigen B27
ILAR: International league of associations for rheumatology
JIA: Juvenile idiopathic arthritis
MIO: Maximum interincisal opening
MRI: Magnetic resonance imaging
NRS: Numeric rating scale
NSAIDS: Non-steroidal anti-inflammatory drugs
PMIO: Passive maximum interincisal mouth opening
SD: Standard deviation
SPSS: Statistical package for the social sciences
RF: Rheumatoid factor
TMD: Temporomandibular disorder
TMJ: Temporomandibular joint
UMC: University medical center
