# Supplementary material for: Mandibular range of motion in children with juvenile idiopathic arthritis with and without clinically established temporomandibular joint involvement and in healthy children; a cross-sectional study
Source: Pediatr Rheumatol Online J. 2021 Jul 3;19:106. doi: 10.1186/s12969-021-00583-5 (PMC8254997; doi:10.1186/s12969-021-00583-5)
Supplement: Supplementary file 1 — Additional file 1. [file 12969_2021_583_MOESM1_ESM.docx]

**Additional file 1 –TMJ screening protocol**

| History: | |  |  |
| --- | --- | --- | --- |
|  | Problems in chewing | Yes | No |
|  | Eating slower than others | Yes | No |
|  | Biting hard food difficult | Yes | No |
|  | Pain while eating | Yes | No |
|  | Limited mouth opening | Yes | No |
| Examination | |  |  |
|  | Limited mouth opening | Yes | No |
|  | Crepitation (audible) | Yes | No |
|  | Pain AMIO | Yes | No |
|  | Deviation AMIO (>2 mm) | Yes | No |
| Inspection | |  |  |
|  | Asymmetry | Yes | No |
|  | Retrognathia | 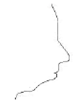  Yes | 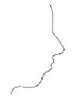  No |

AMIO: active maximum interincisal opening.
The TMJ screening protocol was scored for each child.^5^ The sum of the 11 items was documented. Children with JIA and a TMJ screening protocol score ≥ 2 were labelled as ‘JIA with TMJ involvement’. ‘Audible’ means using a stethoscope for detecting crepitation. Retrognathia was assessed by the examiner following one of three options: normal profile and class II profile ‘no’, and retrognathic profile ‘yes’; the latter two profiles are presented as drawings in the TMJ screening protocol.
