# Supplementary material for: Mandibular range of motion in children with juvenile idiopathic arthritis with and without clinically established temporomandibular joint involvement and in healthy children; a cross-sectional study
Source: Pediatr Rheumatol Online J. 2021 Jul 3;19:106. doi: 10.1186/s12969-021-00583-5 (PMC8254997; doi:10.1186/s12969-021-00583-5)
Supplement: Supplementary file 2 — Additional file 2. [file 12969_2021_583_MOESM2_ESM.docx]

**Additional file 2 – Adjusted linear regression models 1 and 2 for mandibular range of motion in children with JIA, with and without TMJ involvement and healthy children, including age instead of length**

| **Model 1: Children with JIA vs healthy children** | | | | | | | | |
| --- | --- | --- | --- | --- | --- | --- | --- | --- |
|  | **AMIO** | | **PMIO** | | **Protrusion** | | **Discrepancy in laterotrusion:** | |
| Variable | **Regression coefficients**  **(95% CI)** | ***P-value*** | **Regression coefficients**  **(95% CI)** | ***P-value*** | **Regression coefficients**  **(95% CI)** | ***P-value*** | **Regression coefficients**  **(95% CI)** | ***P-value*** |
| JIA vs healthy children | -3.71 (-5.02 – -2.41) | 0.000 | -3.93 (-5.32 – -2.50) | 0.000 | -1.08 (1.50 – -0.65) | 0.000 | 0.67 (0.46–0.89) | 0.000 |
| Male gender | 1.94 (0.67–3.20) | 0.003 | 1.31 (0.02--2.64) | 0.053 | 0.18 (-0.24–0.61) | 0.392 | -0.14 (-0.35–0.07) | 0.184 |
| Age (centered at 6) | 1.37 (0.71–2.03) | 0,000 | 1.53 (0.85–2.22) | 0.000 | 0.32 (0.10–0.54) | 0.005 | -0.02 (-0.13–0.09) | 0.690 |
| Age squared (centered at 6) | -0.06 (-0.11–-0.00) | 0.034 | -0.07 (-0.12 – -0.01) | 0.023 | -0.02 (-0.04 – -0.00) | 0.020 | 0.00 (-0.01–0.01) | 0.565 |
| Intercept | 48.61 (45.99–51.22) |  | 49.59 (46.84–52.33) |  | 8.77 (7.91–9.63) |  | -0.47 (-0.90 – -0.04) |  |
| R^2^ | 0.18 | | 0.18 | | 0.07 | | 0.10 | |
| **Model 2: Children with JIA with vs without TMJ involvement** | | | | | | |  | |
|  | **AMIO** | | **PMIO** | | **Protrusion** | | **Discrepancy in laterotrusion:** | |
| Variable | **Regression coefficients**  **(95% CI)** | ***P-value*** | **Regression coefficients**  **(95% CI)** | **P-value** | **Regression coefficients**  **(95% CI)** | **P-value** | **Regression coefficients**  **(95% CI)** | **P-value** |
| JIA vs healthy children | -1.72 (-3.00 – -0.44) | 0.008 | -2.27 (-3.66 – -0.89) | 0.001 | -0.96 (-1.42 -- -0.50) | 0.000 | 0.57 (0.35–0.80) | 0.000 |
| JIA vs without TMJ involvement | -7.12 (-8.66 – -5.59) | 0.000 | -6.12 (-7.75 – -4.49) | 0.000 | -0.31 (-0.88–0.25) | 0.278 | 0.35 (0.08–0.62) | 0.011 |
| Male gender | 1,78 (0.62–2.95) | 0.003 | 1.16 (-0.10–2.41) | 0.070 | -0.18 (-0.60 – 0.25) | 0.416 | -0.13 (-0.34–0.07) | 0.210 |
| Age (centered at 6) | 1.35 (0.74–1.96) | 0.000 | 1.52 (0.87–2.17) | 0.000 | 0.32 (0.10–0.54) | 0.005 | -0.02 (-0.13–0.09) | 0.706 |
| Age squared (centered at 6) | -0.05 (-0.10–0.01) | 0.075 | -0.05 (-0.11 – -0.00) | 0.048 | -0.02 (-0.04–0.00) | 0.023 | 0.00 (-0.01–0.01) | 0.667 |
| Intercept | 46.10 (43.62–48.56) |  | 47.48 (44.83–50.13) |  | 8.66 (7.77–9.55) |  | -0.35 (-0.79–0.10) |  |
| R^2^ | 0.30 | | 0.27 | | 0.07 | | 0.11 | |

AMIO: active maximum interincisal opening; CI: confidence interval; JIA: juvenile idiopathic arthritis; PMIO: passive maximum interincisal opening; TMJ: temporomandibular joint.
*Discrepancy in laterotrusion was defined as the difference in laterotrusion (in millimeters) between the left and right side. TMJ involvement is proposed as a TMJ protocol score ≥ 2 in JIA patients.
