# Supplementary material for: Mandibular range of motion in children with juvenile idiopathic arthritis with and without clinically established temporomandibular joint involvement and in healthy children; a cross-sectional study
Source: Pediatr Rheumatol Online J. 2021 Jul 3;19:106. doi: 10.1186/s12969-021-00583-5 (PMC8254997; doi:10.1186/s12969-021-00583-5)
Supplement: Supplementary file 3 — Additional file 3. [file 12969_2021_583_MOESM3_ESM.zip › Additional file 3, captions.docx]

**Additional file 3 – Estimated marginal means for AMIO vs age in children with JIA, with and without TMJ involvement and in healthy children**

The estimated marginal means of the AMIO are calculated from the linear regression model of AMIO (Additional file B). The AMIO, in millimeters (mm) for each age (in years), is presented for children with JIA, with and without TMJ involvement, and healthy children.
