# Supplementary material for: Mandibular range of motion in children with juvenile idiopathic arthritis with and without clinically established temporomandibular joint involvement and in healthy children; a cross-sectional study
Source: Pediatr Rheumatol Online J. 2021 Jul 3;19:106. doi: 10.1186/s12969-021-00583-5 (PMC8254997; doi:10.1186/s12969-021-00583-5)
Supplement: Supplementary file 4 — Additional file 4. [file 12969_2021_583_MOESM4_ESM.docx]

**Additional file 4** **– Unadjusted linear regression models for active maximum interincisal opening (AMIO) with the variables temporomandibular joint (TMJ) involvement and corrected TMJ involvement**

|  | **Regression coefficients**  **(95% CI)** | **P-value** | **R^2^** |
| --- | --- | --- | --- |
| TMJ involvement | -6.56 (-8.12 – -5.00) | 0.000 | 0.128 |
| Corrected TMJ involvement | -5.87 (-7.66 – -4.08) | 0.000 | 0.082 |

CI: confidence interval
TMJ involvement is proposed as a TMJ screening protocol score ≥ 2 in children with juvenile idiopathic arthritis (JIA) [5]. The corrected TMJ involvement is presented as: a TMJ screening protocol score ≥ 2 in JIA without the items “limited mouth opening in the medical history,” “limited mouth opening during clinical examination,” and “deviation during active maximum interincisal opening (AMIO).”
