# Supplementary material for: Mandibular range of motion in children with juvenile idiopathic arthritis with and without clinically established temporomandibular joint involvement and in healthy children; a cross-sectional study
Source: Pediatr Rheumatol Online J. 2021 Jul 3;19:106. doi: 10.1186/s12969-021-00583-5 (PMC8254997; doi:10.1186/s12969-021-00583-5)
Supplement: Supplementary file 5 — Additional file 5. [file 12969_2021_583_MOESM5_ESM.docx]

**Additional file 5 – Unadjusted linear regression models for passive maximum interincisal opening (PMIO) with the variables temporomandibular joint (TMJ) involvement and corrected TMJ involvement**

|  | **Regression coefficients**  **(95% CI)** | **P-value** | **R^2^** |
| --- | --- | --- | --- |
| TMJ involvement | -5.40 (-7.05 – -3.74) | 0.000 | 0.085 |
| Corrected TMJ involvement | -4.89 (-6.77 – -3.02) | 0.000 | 0.054 |

CI: confidence interval.

TMJ involvement is proposed as a TMJ screening protocol score ≥ 2 in children with juvenile idiopathic arthritis (JIA) [5]. The corrected TMJ involvement is presented as: a TMJ screening protocol score ≥ 2 in JIA without the items “limited mouth opening in the medical history,” “limited mouth opening during clinical examination,” and “deviation during active maximum interincisal opening (AMIO)”.
